# Successful Treatment of Disseminated Nocardiosis by Rapid Identification of the Organism via Genetic Analysis in a Leukemia Patient Undergoing Allogeneic Hematopoietic Stem Cell Transplantation

**DOI:** 10.7759/cureus.58489

**Published:** 2024-04-17

**Authors:** Tomoe Anan, Yasuyuki Takahashi, Yuta Kimura, Takayuki Tabayashi, Yasushi Kubota

**Affiliations:** 1 Department of Hematology, Saitama Medical University, Saitama Medical Center, Kawagoe, JPN

**Keywords:** 16s ribosomal rna, genetic analysis, allogeneic hematopoietic stem cell transplantation, disseminated nocardiosis, nocardia

## Abstract

*Nocardia* infections have been reported to occur in immunocompromised patients. Early diagnosis and therapeutic intervention are especially important for disseminated nocardiosis because of its high mortality rate. A case of disseminated nocardiosis after allogeneic hematopoietic stem cell transplantation, which was promptly treated after identification of the organism by genetic analysis, is presented. A 43-year-old man was diagnosed with T-cell prolymphocytic leukemia and underwent allogeneic hematopoietic stem cell transplantation. Subsequently, during long-term prednisolone administration for chronic graft-versus-host disease, he developed mass lesions throughout his body at 1033 days after transplantation. Pus culture and genetic testing of the parotid mass showed *Nocardia farcinica*, which improved with treatment with sulfamethoxazole, trimethoprim, and imipenem cilastatin, and there has been no recurrence. When multiple mass lesions occur after hematopoietic stem cell transplantation, and the diagnosis is difficult, disseminated nocardiosis should be included in the differential diagnosis, and appropriate laboratory testing and treatment should be performed.

## Introduction

Nocardiosis is an infectious disease caused by gram-positive aerobic actinomycetes, which can affect the skin, respiratory tract, central nervous system, and other organs [1-3]. Although relatively rare, approximately 60-70% of nocardiosis cases are reported to occur in immunocompromised patients [4,5]. There are scattered reports of nocardiosis being diagnosed after hematopoietic stem cell transplantation (HSCT) in other countries, but few in Japan [3,6,7]. One of the major complications of allogeneic HSCT is graft-versus-host disease (GVHD), which can present with a variety of symptoms [8]. Infections, including nocardiosis, can also sometimes be systemic in their manifestations. However, GVHD and infections each require different forms of treatment. Therefore, differential diagnosis is extremely important. Early diagnosis and therapeutic intervention are critical because of the high mortality rate of disseminated nocardiosis [3,9], but identification of the organism is still difficult.

A case of disseminated nocardiosis after allogeneic HSCT, in which genetic analysis was used to identify the organism, and appropriate treatment could be selected, is presented.

## Case presentation

A 43-year-old man was diagnosed with T-cell prolymphocytic leukemia (T-PLL). He received allogeneic HSCT from an human leukocyte antigen (HLA)-matched related donor. The number of CD34-positive cells infused was 3.76×10^6^/kg and neutrophil engraftment was achieved at Day 17 after transplant. The GVHD prophylaxis included cyclosporine (CsA) and methotrexate (MTX) at a dose of 5 mg/m^2^ on Days 1, 3 and 6, and no acute GVHD was observed. CsA was discontinued on Day 110 due to renal dysfunction possibly caused by CsA. At the same time, prednisolone (PSL) 60 mg/day (1 mg/kg) was started for liver dysfunction and an oral mucosal disorder possibly caused by chronic GVHD. The symptoms improved, but it took three years to taper PSL to 10 mg/day. The combination of sulfamethoxazole and trimethoprim (TMP-SMX) was given at a dose of 480 mg once daily, two days a week, to prevent Pneumocystis jirovecii pneumonia. Renal dysfunction appears to be primarily a side effect of CsA. He also had diabetes mellitus associated with steroid use.

Three years after allogeneic HSCT, scleroderma-like lesions were observed on the patient’s lower back and abdomen. Chronic GVHD was diagnosed and treated with increased doses of PSL (50 mg/day) and mycophenolate mofetil (MMF), but it was refractory to treatment. Furthermore, rituximab was added, but the symptoms did not improve. Subsequently, he became aware of a parotid mass, pain in his right eye, and polyopia. The patient was admitted to our hospital for close examination and treatment.

On admission, the patient had a fever (37.6 °C) and was slightly hypertensive (136/92 mmHg), but his pulse rate was normal (75 bpm). On physical examination, diplopia during upward gaze was seen along with right eye pain. A 2-cm mass with redness was palpable in the right parotid region. Breath sounds were clear. Subcutaneous induration and sclerema were present in the abdomen and both lower legs. There was swelling with heat on the extensor aspect of the right thigh, and superficial lymph nodes were not palpable. Laboratory tests on admission showed a leukocyte count of 8.3×10^9^/L (normal range 3.3-8.6×10^9^), with markedly increased neutrophils (94%), and the lymphocyte count was 249/L. The hemoglobin concentration was 10.3 g/L (normal range 13.7-16.8), and the platelet count was 135×10^9^/L (normal range 158-348). On blood chemistry analysis, the serum C-reactive protein (CRP) concentration was 2.5 mg/dL (normal range 0.00-0.14), and he had hypogammaglobulinemia [IgG 3.30 g/L (normal range 8.61-17.47), IgA 0.20 g/L (0.93-3.93), and IgM 0.20 g/L (0.33-1.83)]. Procalcitonin, β-D-glucan, and aspergillus antigen were all negative (Table 1).

**Table 1:** Laboratory findings on admission WBC, white blood cell; Stab, stab neutrophil; Seg, segmented neutrophil; Mono, monocyte; Lymph, lymphocyte; RBC, red blood cell; Hb, hemoglobin; Ht, hematoclit; MCV, Mean corpuscular volume; APTT, activated partial thromboplastin time; PT-INR, prothrombin time-international normalized ratio; TP, total protein; Alb, albumin; AST, aspartate aminotransferase; ALT, alanine aminotransferase; LDH, lactate dehydrogenase; BUN, blood urea nitrogen; Cre, creatinine; UA, uric acid; Na, sodium; Cl, chlorine; K, potassium; T-Bil, total bilirubin; CRP, C-reactive protein; IgG, immunoglobulin G; IgA, immunoglobulin A; IgM, immunoglobulin M

Parameters	Values	Units
Complete blood count		
WBC	8.3×10^9^	/L
Stab	14	%
Seg	80	%
Mono	3	%
Lymph	3	%
RBC	3.08×10^12^	/L
Hb	10.3	g/L
Ht	30.8	%
MCV	100	fL
Platelets	135×10^9^	/L
Coagulation		
APTT	32.3	sec
PT-INR	0.79	
Fibrinogen	638	mg/dL
Biochemistry		
TP	5.9	g/dL
Alb	3.3	g/dL
AST	18	U/L
ALT	26	U/L
LDH	494	U/L
BUN	57	mg/dL
Cr	2.17	mg/dL
UA	5.6	mg/dL
Na	140	mEq/L
Cl	100	mEq/L
K	4.4	mEq/L
T-Bil	0.9	mg/dL
CRP	2.5	mg/dL
Immunology		
IgG	3.3	g/L
IgA	0.2	g/L
IgM	0.2	g/L
Procalcitonin	negative	
β-D-glucan	negative	
Aspergillus antigen	negative	

**Figure 1:**
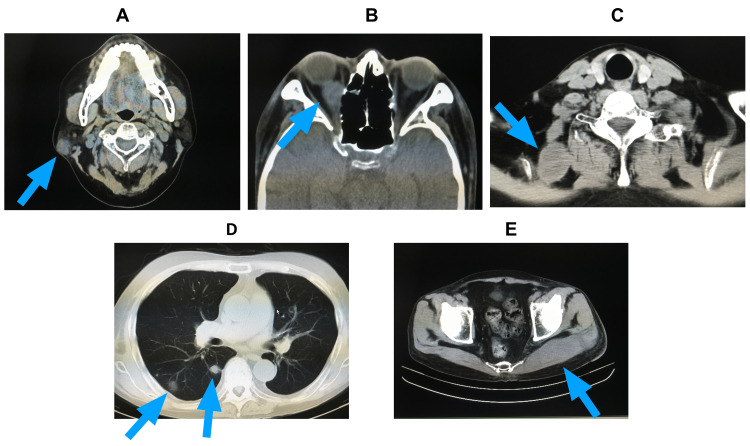
Computed tomography findings on admission. In addition to mass lesions in the right parotid gland area (A) and right orbit (B), the image shows swelling of the right levator scapulae muscle (C) and left gluteal muscle (E). Multiple pulmonary nodules (D) can also be seen.

Computed tomography (CT) showed masses in the right parotid region, right orbit, right scapula, and left gluteal region. Multiple pulmonary nodules were also found (Figure 1).

**Figure 2:**
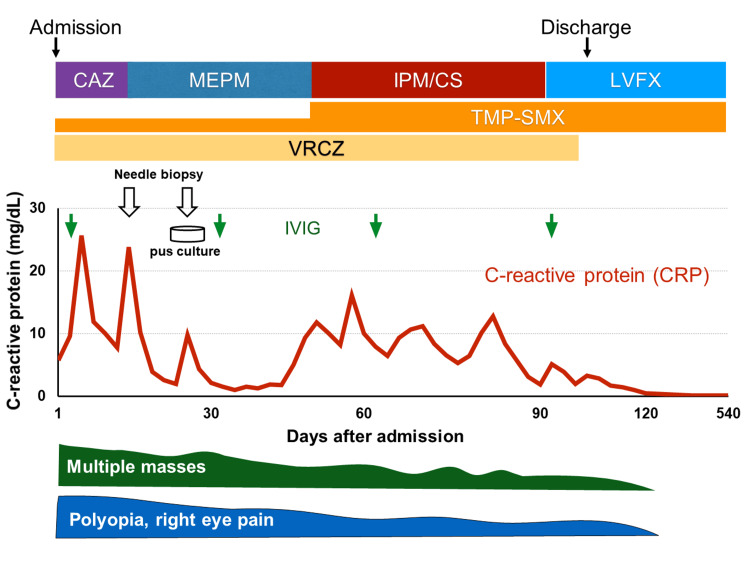
The clinical course after admission. Improvement of symptoms derived from disseminated nocardiosis and changes in C-reactive protein (CRP) levels are shown. CAZ: ceftazidime, MEPM: meropenem, IPM/CS: imipenem cilastatin, TMP-SMX: sulfamethoxazole and trimethoprim, VRCZ: voriconazole, LVFX: levofloxacin, IVIG: intravenous high-dose immunoglobulin therapy

After admission, in addition to diplopia and right eye pain, pain in the left thigh worsened, and walking became difficult. CRP increased to 25.7 mg/dL along with high fever. Needle biopsies of the right levator scapulae and left gluteal muscles were performed 14 days after admission for close examination. Pathology results were class II, with a neutrophilic inflammatory picture with eosinophils. The results ruled out the recurrence of leukemia but did not identify a cause of infection. Since multiple nodular shadows were observed in the lungs, fungal infection was suspected, and voriconazole (VRCZ) was administered. The possibility of bacterial infection was also considered, and ceftazidime (CAZ) and meropenem (MEPM) were given. This resulted in the resolution of the fever and reduction of pain in the left thigh several days later, and CRP decreased to 2.0 mg/dL (Figure 2).

A needle biopsy of the mass in the left parotid region performed 26 days after admission showed drainage of pus, and culture of the pus showed branched gram-positive bacilli, which led to suspicion of nocardiosis.

**Figure 3:**
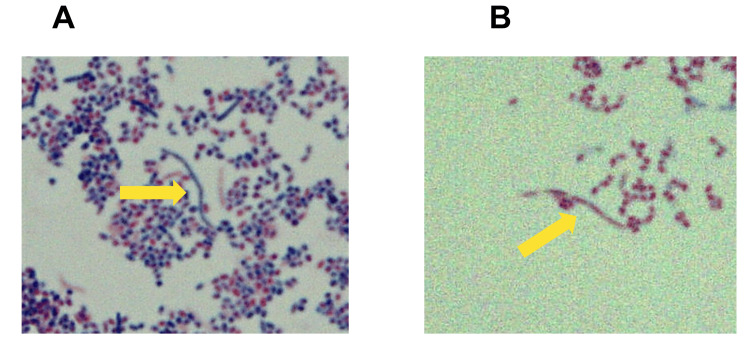
Stained image of pus. A puncture culture from a right parotid mass shows branched gram-positive rods (A), which are positive for Kinyoun staining (B) and suspected to be *Nocardia spp.* (original magnification ×1000).

To identify the species of *Nocardia*, molecular biologic analysis was performed using 16S ribosomal RNA (16S rRNA) gene sequencing, and the 16S rRNA sequence was determined by direct sequencing of polymerase chain reaction (PCR) products. The obtained 1,482 bp sequences were analyzed using EzTaxon, resulting in two strains with more than 99.0% 16S rRNA sequence similarity (*Nocardia* (*N*)* kroppenstedtii*): 99.93%, *N.* *farcinica*: 99.86%]. Although the 16S rRNA-based classification could not classify the above two strains, they could be differentiated by their growth at 37 °C. *N. kroppenstedtii* is characterized by growth at 37 °C, whereas *N. farcinica* is characterized by no growth at 37 °C. The strain obtained from the pus culture was identified as *N. farcinica* because it did not grow at 37 °C (Figure 3).

The only target organ of the patient's chronic GVHD was the skin, which was predominantly sclerosing changes and lichen planus-like rash. The severity of his chronic GVHD was moderate, with a score of 2 (around 30%) as calculated by the percentage of the body surface area. At the time, the PSL dose was increased in anticipation of possible improvement with immunosuppressive drugs, but in retrospect, the progression of the disease was presumably irreversible, and prompt reduction of the PSL dose after the diagnosis of disseminated nocardiosis did not result in exacerbation of chronic GVHD.

CRP elevation and high fever, which had been relieved by MEPM and VRCZ, worsened again. TMP-SMX (960 mg daily) and imipenem cilastatin (IPM/CS) were started. Despite occasional fever and elevated CRP, the lesions gradually shrank on CT, and ocular pain and diplopia improved after about three months of TMP-SMX and IPM/CS treatment. After discontinuation of IPM/CS, the patient was switched to taking TMP-SMX and levofloxacin (LVFX) (Figure 2). After one year and two months of continuous treatment following the diagnosis of disseminated nocardiosis, the drug was discontinued, and there has been no recurrence.

## Discussion

Herein, we describe a case of disseminated nocardiosis after allogeneic HSCT in which 16S rRNA sequencing was used to identify the organism, which facilitated the selection of appropriate antibiotics and cure of disseminated nocardiosis.

*Nocardia* are aerobic gram-positive rods belonging to the actinomycetes order Nocardiaceae, and they are widely distributed in soil. *N. asteroides* is frequently isolated from clinical specimens, but *N. farcinica, N. nova, N. brasiliensis, *and* N. otitidiscaviarum* have also been isolated as causative organisms [1,2]. Of *Nocardia spp.*, *N. farcinica* is the most virulent, and it is reported to cause disseminated lesions in the brain and other parts of the body [1,3]. The mortality rate of disseminated nocardiosis due to *N. farcinica* is as high as 39% [10]. The respiratory tract is the most common site of infection, but infection can occur in any organ, including the skin, central nervous system, and soft tissues [3,11]. Because lesions present with various findings on imaging, including infiltrative shadows, nodular/tumor shadows, and abscess patterns, it is difficult to distinguish the species of bacteria based on clinical symptoms and imaging findings alone [3,12].

When there was a strong focus on orbital lesions prior to the diagnosis of nocardiosis in the present case, the differential diagnosis was recurrence of T-PLL as lymphomatous lesions or the development of post-transplant lymphoproliferative disease, although atypical in timing. If so, the treatment of choice would likely have been early reduction of immunosuppressive drugs or donor lymphocyte infusion, which would have exacerbated the GVHD. Diagnosis requires detection of the bacteria at the site of infection, but *Nocardia spp.* are slow-growing, requiring several weeks in culture, and are often missed because they are decolorized by the usual Ziehl-Neelsen staining [13]. An important test in diagnosis is the identification of a branched form of the organism that is positive for Gram and Kinyoun staining of specimens collected from the lesion site, but recently genetic testing using 16S rRNA has enabled early confirmation of the diagnosis [14].

Since the susceptibility to antimicrobial drugs varies with the species, the identification of causative organisms and their susceptibility is crucial in the treatment of the disease. Regarding the treatment of *Nocardia* infections, TMP-SMX is the first-line therapy and the therapeutic dose of TMP-SMX for disseminated nocardiasis is 15 mg/kg TMP equivalent. The duration of treatment is usually 6-12 months, with brain abscesses and immunocompromised states requiring a minimum of 12 months of administration. In cases where a high mortality rate is expected, combination therapy may be used with TMP-SMX and amikacin, MEPM, IPM/CS, or ceftriaxone, etc. [15,16]. In the present case, IPM/CS and LVFX were used in addition to TMP-SMX for the treatment of disseminated nocardiosis, resulting in no subsequent recurrence.

## Conclusions

A case of disseminated nocardiosis after allogeneic HSCT in which the organism was identified by 16S rRNA sequencing and cured with antibiotics was reported. Because of the high mortality rate of disseminated nocardiosis, early definitive diagnosis and therapeutic intervention are important, but imaging diagnosis is difficult. Genetic testing using 16S rRNA is useful for early diagnosis and treatment. Accurate identification of the species and susceptibility testing should be performed whenever possible since antimicrobial susceptibility varies depending on the bacterial species. Nocardiosis should also be kept in mind when multiple lesions are present in patients after HSCT and diagnosis is difficult.

## References

[1] Tremblay J, Thibert L, Alarie I, Valiquette L, Pépin J (2011). Nocardiosis in Quebec, Canada, 1988-2008. Clin Microbiol Infect.

[2] Ambrosioni J, Lew D, Garbino J (2010). Nocardiosis: updated clinical review and experience at a tertiary center. Infection.

[3] Averbuch D, De Greef J, Duréault A (2022). Nocardia infections in hematopoietic cell transplant recipients: a multicenter international retrospective study of the Infectious Diseases Working Party of the European Society for Blood and Marrow Transplantation. Clin Infect Dis.

[4] Coussement J, Lebeaux D, Rouzaud C, Lortholary O (2017). Nocardia infections in solid organ and hematopoietic stem cell transplant recipients. Curr Opin Infect Dis.

[5] Lebeaux D, Freund R, van Delden C (2017). Outcome and treatment of nocardiosis after solid organ transplantation: new insights from a European study. Clin Infect Dis.

[6] Shannon K, Pasikhova Y, Ibekweh Q, Ludlow S, Baluch A (2016). Nocardiosis following hematopoietic stem cell transplantation. Transpl Infect Dis.

[7] Yamakawa H, Yoshida M, Morikawa N (2014). Pulmonary Nocardia nova infection after allogeneic hematopoietic stem cell transplantation. Intern Med.

[8] Zeiser R, Blazar BR (2017). Pathophysiology of chronic graft-versus-host disease and therapeutic targets. N Engl J Med.

[9] Malincarne L, Marroni M, Farina C (2002). Primary brain abscess with Nocardia farcinica in an immunocompetent patient. Clin Neurol Neurosurg.

[10] Budzik JM, Hosseini M, Mackinnon AC Jr, Taxy JB (2012). Disseminated Nocardia farcinica: literature review and fatal outcome in an immunocompetent patient. Surg Infect (Larchmt).

[11] Wilson JW (2012). Nocardiosis: updates and clinical overview. Mayo Clin Proc.

[12] Beaman BL, Beaman L (1994). Nocardia species: host-parasite relationships. Clin Microbiol Rev.

[13] Matsuo K, Takeuchi M, Kawata N (2000). [Pulmonary Nocardia otitidiscaviarum infection in an immunocompetent host]. Nihon Kokyuki Gakkai Zasshi.

[14] Cloud JL, Conville PS, Croft A, Harmsen D, Witebsky FG, Carroll KC (2004). Evaluation of partial 16S ribosomal DNA sequencing for identification of nocardia species by using the MicroSeq 500 system with an expanded database. J Clin Microbiol.

[15] Corti ME, Villafañe-Fioti MF (2003). Nocardiosis: a review. Int J Infect Dis.

[16] Brown-Elliott BA, Brown JM, Conville PS, Wallace RJ Jr (2006). Clinical and laboratory features of the Nocardia spp. based on current molecular taxonomy. Clin Microbiol Rev.

